# Viruses of Apple Are Seedborne but Likely Not Vertically Transmitted

**DOI:** 10.3390/v16010095

**Published:** 2024-01-07

**Authors:** Anna Wunsch, Bailey Hoff, Mario Miranda Sazo, Janet van Zoeren, Kurt H. Lamour, Oscar P. Hurtado-Gonzales, Marc Fuchs

**Affiliations:** 1 Plant Pathology and Plant-Microbe Biology Section, School of Integrative Plant Science, Cornell University, Geneva, NY 14456, USA; bkhoff7@gmail.com (B.H.); marc.fuchs@cornell.edu (M.F.); 2 Biology Department, Gustavus Adolphus College, St. Peter, MN 56082, USA; 3 Cornell Cooperative Extension Lake Ontario Fruit Program, Albion, NY 14411, USA; mrm67@cornell.edu (M.M.S.); jev67@cornell.edu (J.v.Z.); 4 Department of Entomology and Plant Pathology, University of Tennessee, Knoxville, TN 37996, USA; klamour@utk.edu; 5 USDA-APHIS Plant Germplasm Quarantine Program, Beltsville, MD 20705, USA; oscar.hurtado-gonzales@usda.gov

**Keywords:** apple, *Malus domestica*, latent virus, seed transmission, seedborne virus, apple chlorotic leaf spot virus, apple stem grooving virus, apple stem pitting virus

## Abstract

Many viruses occur in apple (*Malus domestica* (Borkh.)), but no information is available on their seed transmissibility. Here, we report that six viruses infecting apple trees, namely, apple chlorotic leaf spot virus (ACLSV), apple green crinkle-associated virus (AGCaV), apple rubbery wood virus 2 (ARWV2), apple stem grooving virus (ASGV), apple stem pitting virus (ASPV), and citrus concave gum-associated virus (CCGaV) occur in seeds extracted from apple fruits produced by infected maternal trees. Reverse transcription polymerase chain reaction (RT-PCR) and quantitative RT-PCR (RT-qPCR) assays revealed the presence of these six viruses in untreated apple seeds with incidence rates ranging from 20% to 96%. Furthermore, ASPV was detected by RT-PCR in the flesh and peel of fruits produced by infected maternal trees, as well as from seeds extracted from apple fruits sold for fresh consumption. Finally, a large-scale seedling grow-out experiment failed to detect ACLSV, ASGV, or ASPV in over 1000 progeny derived from sodium hypochlorite surface sterilized seeds extracted from fruits produced by infected maternal trees, suggesting no detectable transmission via embryonic tissue. This is the first report on the seedborne nature of apple-infecting viruses.

## 1. Introduction

The domestic apple, *Malus domestica* (Borkh.), is one of the most widely grown tree fruit crops in the world [1]. Of the more than 30 viruses and viroids that have been reported to infect apple trees, several are distributed worldwide and occur wherever apples are cultivated [2,3]. The most common apple viruses include apple chlorotic leaf spot virus (ACLSV), apple stem grooving virus (ASGV), and apple stem pitting virus (ASPV), all of which are members of the family *Betaflexiviridae* [2,3]. Other viruses and viroids of apple trees include the foveavirus apple green crinkle-associated virus (AGCaV), the ilarvirus apple mosaic virus (ApMV), the nepoviruses tobacco ringspot virus (TRSV) and tomato ringspot virus (ToRSV), and the apscaviroids apple dimple fruit viroid (ADFVd), apple fruit crinkle viroid (AFCVd), apple scar skin viroid (ASSVd), and pear blister canker viroid (PBCVd). In recent years, advancements in sequencing-based diagnostic techniques have enabled the identification of many novel or putative systemic pathogens, including viruses and viroids. These include the ilarvirus apple ilarvirus 2 [4], the luteovirus apple luteovirus 1 (ALV1) [5], the rubodviruses apple rubbery wood virus 1 (ARWV1) and apple rubbery wood virus 2 (ARWV2) [6], the coguviruses citrus concave gum-associated virus (CCGaV) [7] and citrus virus A (CiVA) [8], the tepovirus Prunus virus T (PrVT) [9], the pelamoviroid apple hammerhead viroid (AHVd) [10,11], and others [12]. Many of the novel viruses and viroids recently identified via high-throughput sequencing have yet to be biologically characterized, and therefore little is known about their transmission.

Surprisingly, given the wide distribution of many apple viruses and viroids, only two apple-infecting viruses (TRSV and ToRSV) have been reported to be transmitted by vectors [2,13,14], and no virus and only one viroid, ASSVd, has ever been reported to be seedborne or seed transmitted in apple [2,15]. Instead, most apple viruses and viroids have historically been believed to be transmitted only through vegetative propagation of their hosts, including via grafting and budding, but it remains unclear how their plant-to-plant transmission occurred prior to apple cultivation. Mechanical inoculation of herbaceous hosts has been achieved experimentally for ACLSV, ApMV, ASGV, ASPV, ToRSV, and PBCVd, but is not believed to contribute to natural transmission in orchard settings [2]. Additionally, transmission of viruses via root grafting has been suggested as a possible route of natural transmission, but definitive evidence has yet to be produced. 

Though pome fruit crops are primarily vegetatively propagated, seed propagation plays a critical role in the development of new scion cultivars and rootstock genotypes in apple breeding programs [16]. Seeds are also used to facilitate the global distribution of germplasm, the establishment of germplasm repositories, and the production of seedling rootstocks [17,18,19,20]. Although extensive phytosanitary regulations have been established in many countries to limit the spread of systemic pathogens of apple via the import of apple clonal propagating material, seeds have often been exempted from these regulations because no major viruses of pome fruit crops have ever been reported to be seedborne or seed transmitted. Pome fruit seeds are therefore typically considered at a low risk for facilitating virus dissemination [17,18]. However, a recent report documented that ACLSV, ASGV, and ASPV are both seedborne and seed transmitted in two pear rootstock species, *Pyrus betulifolia* and *P. calleryana*, wherein the three viruses were detected in both seeds and progeny seedlings of infected maternal trees [21]. These findings demonstrated that ACLSV, ASGV, and ASPV are seedborne in these host species, i.e., they contaminate or infect the seedcoats of the seeds extracted from fruits produced by infected maternal trees; however, it remains unclear whether any of these viruses are present in embryonic tissue in these species. Regardless, the detection of these three viruses in progeny seedlings suggests that they are indeed capable of being transmitted to progeny of infected *P. betulifolia* and *P. calleryana*, whether this occurs via direct virus invasion of the embryo or via infection of the seedlings during germination.

In light of the importance of apple seed propagation and the potential phytosanitary risks posed by apple-infecting viruses and viroids, the current study was conducted to evaluate whether some viruses of pome fruit crops (i.e., ACLSV, AGCaV, ARWV2, ASGV, ASPV, and CCGaV) are seedborne or seed transmitted in commercial cultivars of *M. domestica*. We hypothesized that these six viruses could be both seedborne and seed transmitted. Here, we report that all six of these viruses are indeed seedborne, though no evidence of seedling infection via seed transmission was observed.

## 2. Materials and Methods

### 2.1. Experimental Apple Orchard Establishment and Fruit Collection

**Figure 1:**
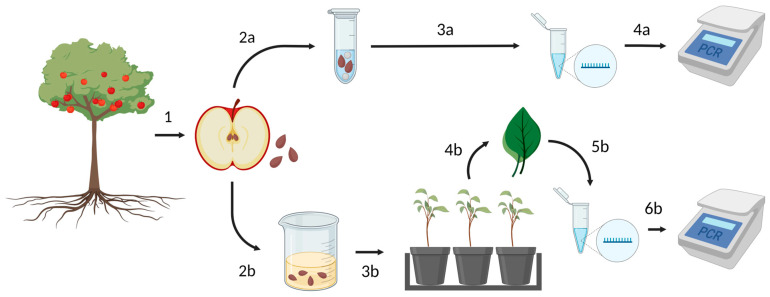
Experimental workflow for evaluating the seedborne incidence and seed transmission of apple chlorotic leaf spot virus (ACLSV), apple green crinkle-associated virus (AGCaV), apple rubbery wood virus 2 (ARWV2), apple stem grooving virus (ASGV), apple stem pitting virus (ASPV), and citrus concave gum-associated virus (CCGaV) in *Malus domestica*. (1) Representative fruits resulting from the open pollination of virus-infected maternal trees were collected from an experimental orchard and seeds were extracted from each fruit to evaluate ACLSV, AGCaV, ARWV2, ASGV, ASPV, and CCGaV detectability in seedcoats. (2a) Two untreated, intact seeds from each virus-infected maternal tree were disrupted in lysis buffer with steel beads (gray circles) to disrupt the seedcoats without fragmenting the seeds. (3a) Total RNA was isolated from the resulting lysate and was then (4a) screened for ACLSV, AGCaV, ARWV2, ASGV, ASPV, and CCGaV by reverse transcription (RT) polymerase chain reaction (PCR) assays. To evaluate seed transmission of ACLSV, ASGV, and ASPV from virus-infected maternal trees to their progeny, (2b) seeds were surface sterilized with sodium hypochlorite and cold stratified. (3b) Seeds were then germinated and grown under greenhouse conditions until they reached the 6–8 true leaf stage, at which point (4b) leaf tissue was collected from each seedling for nucleic acid isolation. (5b) Total RNA was isolated from each leaf tissue sample and (6b) screened for ACLSV, ASGV, and ASPV by RT-PCR assays. Created with BioRender.com.

An experimental high-density apple orchard block was established in 2019 on a well-drained loam soil (Lima series) at Cornell AgriTech in Ontario County, NY, USA [22]. This orchard was originally designed to study the potential role of viruses in the apple decline phenomenon. Trees representing six scion–rootstock combinations (‘Brookfield Gala’, ‘Honeycrisp’, and ‘Royal Red Honeycrisp’ on ‘Malling 26′ or ‘Geneva 935′ rootstocks) were established at 0.9 m × 3.4 m high-density spacing with trellis support and drip irrigation. Trees were maintained according to standard horticultural and pest management practices for conventional apple production in the region. Ten mature fruits resulting from open pollination were collected from each of the 175 trees in August–September 2022 and stored at 4 °C for up to 14 days prior to seed extraction (Figure 1).

### 2.2. Apple Seed Collection and Germination

Seeds were manually extracted from apple fruits collected from each tree in the experimental orchard. Approximately ten seeds from each tree, comprising one seed from each of ten representative fruits, were stored at −80 °C prior to RNA extraction. The remaining seeds were air dried at room temperature for 24 h, treated with 0.75% sodium hypochlorite for 30 min, thoroughly rinsed with distilled water, and soaked in distilled water for 12 h. Treated seeds were then stratified for 12 weeks in the dark at 4 °C. During seed stratification, seeds were kept moist with distilled water, and any seeds showing signs of microbial contamination were discarded. Stratified seeds were sown in coarse seed-starting potting media (LM-GPS Germination Mix, Lambert, Rivière-Ouelle, QC, Canada) and grown under greenhouse conditions (photoperiod: 14 h light/10 h dark; temperature: 22 °C day/20 °C night). After 21 days, the germination rate was evaluated, and seedlings were transplanted to potting soil and fertilized with a 14-14-14 slow-release fertilizer. Transplant viability was evaluated after an additional seven days.

### 2.3. Virus Detection in Apple Trees

To determine the virus infection status of each of the 175 maternal trees in the experimental orchard, a composite leaf tissue sample consisting of 5–6 leaves from throughout the scaffold per tree and a composite root tissue sample consisting of fine roots excavated from the top six inches of soil surrounding the trunk per tree were collected in summer 2022. Then, approximately 100 mg of each tissue sample was homogenized in a guanidine thiocyanate and β-mercaptoethanol lysis buffer with two steel beads (4.5 mm diameter) using a Retsch MM400 mixer mill (Retsch, Haan, Germany) at 30 Hz for 70 s (leaf tissue) or 180 s (root tissue). Next, total RNA was isolated using the GenCatch Plant Total RNA Miniprep Kit (Epoch Life Science, Missouri City, TX, USA) following the manufacturer’s guidelines, quantified using a Qubit 2.0 Fluorometer (ThermoFisher Scientific, Waltham, MA, USA), and stored at −80 °C for up to three weeks prior to virus screening. Each sample was tested for the presence of 19 viruses and viroids of pome fruit trees (Table 1) via multiplex PCR-based amplicon sequencing, as previously described by Costa et al. [23].

### 2.4. Virus Detection in Apple Seeds

Two untreated seeds from each tree in the experimental orchard were homogenized in guanidine thiocyanate lysis buffer with two steel beads (4.5 mm diameter) using a Retsch MM400 mixer mill at 30 Hz for 180 s, disrupting the seedcoat surface without fragmenting the seeds. Total RNA was then isolated from the resulting lysate using the MagMAX™ Viral RNA Isolation Kit (ThermoFisher Scientific, Waltham, MA, USA) with the KingFisher™ Flex automated extraction instrument (ThermoFisher Scientific, Waltham, MA, USA) following the manufacturers’ guidelines and stored at −80 °C for up to four weeks prior to virus screening. Each sample was screened for ACLSV, ASGV, and ASPV by three multiplex reverse transcription (RT) polymerase chain reaction (PCR) assays using specific primer pairs (Table 2) targeting a 677, 273, or 370 bp coat protein gene fragment, respectively, as previously described [24]. Specific primers targeting a 181 bp plant mRNA (NADH dehydrogenase subunit 5) fragment were included in each RT-PCR assay as an internal control to assess the quality of the total RNA preparations and allow the lack of virus target amplification in an uninfected sample to be distinguished from an assay failure, as previously described [24]. Reactions were carried out with the OneStep Ahead RT-PCR Kit (Qiagen, Carlsbad, CA, USA) in a C1000 Touch Thermal Cycler (Bio-Rad, Hercules, CA, USA) following the manufacturers’ guidelines. Each reaction (12.5 µL final volume) included 1 µL of RNA and final primer concentrations of 800 nM. Thermocycling conditions were as follows: a reverse transcription step at 50 °C for 10 min; an initial denaturation step at 95 °C for 5 min; 35 cycles of 94 °C for 30 s, 62 °C for 30 s, and 72 °C for 1 min; and a final extension at 72 °C for 7 min. DNA amplification products were resolved by electrophoresis on 1.5% agarose gels, stained with GelRed™ (Biotium, Fremont, CA, USA), and visualized under ultraviolet light. Additionally, each sample was tested in duplicate for AGCaV, ARWV2, and CCGaV by quantitative RT-PCR (RT-qPCR) assays using specific primers and probes (Table 2). Reactions were carried out with the TaqMan™ Fast Virus 1-Step Master Mix for qPCR (ThermoFisher Scientific, Waltham, MA, USA) in a QuantStudio™ 7 Pro Real-Time PCR System (ThermoFisher Scientific, Waltham, MA, USA) following the manufacturers’ guidelines. Each reaction (10 µL final volume) included 2 µL of RNA and final primer and probe concentrations of 900 and 250 nM, respectively. Thermocycling conditions were as follows: a reverse transcription step at 50 °C for 5 min; an initial denaturation step at 95 °C for 20 s; and 40 cycles of 95 °C for 3 s and 60 °C for 30 s. A negative control (total RNA from leaves of an uninfected apple tree), a positive control (total RNA from leaves of an infected apple tree), and a non-template control (nuclease-free water) were included in each assay. Seedborne incidence was evaluated as the percentage of seed samples in which each virus was detected.

To determine whether ASPV could be detected in the seeds of apple fruits produced in New York State and sold for fresh consumption, ‘Gala’ apple fruits were purchased in June 2023 from a local grocery store. Seeds were manually extracted from four fruits. Half the seeds from each fruit were air dried at room temperature for 24 h, treated with 0.75% sodium hypochlorite for 30 min, thoroughly rinsed with distilled water, and soaked in distilled water for 12 h. The other half of the seeds from each fruit were untreated. Whole untreated and treated seeds from each fruit were disrupted, as described above, and total RNA was then isolated from the resulting lysate and tested for ASPV by a multiplex RT-PCR assay. Reactions were carried out and analyzed as aforementioned.

### 2.5. ASPV Detection in Apple Fruits

To determine whether ASPV could be detected in apple fruit tissue collected from infected maternal trees in the experimental orchard, fruits were collected in June 2022 from four trees in the experimental orchard known to be infected with ASPV. The peel, flesh, and immature seeds of each fruit were manually separated. Approximately 100 mg of each tissue type was homogenized, and total RNA was then isolated as described above for seeds. Each sample was tested in duplicate for ASPV by RT-qPCR using primers and a probe (Table 2) targeting a coat protein gene fragment as previously described [26]. Reactions were carried out with the SuperScript™ III Platinum One-Step qRT-PCR Kit (ThermoFisher Scientific, Waltham, MA, USA) in a CFX96 Touch Real-Time PCR Detection System (Bio-Rad) following the manufacturers’ guidelines. Each reaction (20 µL final volume) included 2 µL of RNA and final primer and probe concentrations of 600 and 200 nM, respectively. Thermocycling conditions were as follows: a reverse transcription step at 50 °C for 15 min; an initial denaturation step at 94 °C for 2 min; and 40 cycles of 94 °C for 10 s and 58 °C for 1 min. Negative controls (total RNA from leaves of an uninfected apple tree), positive controls (total RNA from leaves of an infected apple tree), and non-template controls (nuclease-free water) were included in duplicate in the assay.

### 2.6. Virus Detection in Apple Seedlings

Once seedlings grown from apple seeds reached the 6–8 true leaf stage, a leaf tissue sample consisting of the first and sixth true leaves was collected from each seedling and stored at −20 °C prior to RNA extraction. Approximately 100 mg of each leaf tissue sample was homogenized in guanidine thiocyanate lysis buffer for approximately five seconds using a HOMEX 7 homogenizing system (BIOREBA, Reinach, Switzerland) set to 70% power. Total RNA was then isolated from the resulting lysate using the MagMAX™ Viral RNA Isolation Kit with the KingFisher™ Flex automated extraction instrument following the manufacturers’ guidelines and stored at −80 °C for up to eight weeks prior to virus screening. Total RNA extracts from individual seedlings representing progeny of unique maternal trees were pooled to create composite samples comprised of the total RNA of 16 seedlings each. These composite samples were screened for ACLSV, ASGV, and ASPV by three multiplex RT-PCR assays and analyzed as described above. A negative control (composite total RNA from leaves of 16 uninfected apple trees), a positive control (composite total RNA from leaves of 15 uninfected apple trees and one infected apple tree), and a non-template control (nuclease-free water) were included in each assay. The seed transmission rate was evaluated as the percent of seedlings in which each virus was detected.

## 3. Results

### 3.1. Detection of Viruses and Viroids in Maternal Apple Trees by Multiplex PCR-Based Amplicon Sequencing

Diagnostic testing of 175 apple trees for 19 viruses and viroids of apple trees via multiplex PCR-based amplicon sequencing revealed the presence of a total of eight viruses and one viroid in the samples tested. At least one virus or viroid was found in every tree tested (Table 3). The three most prevalent viruses were ASGV, ACLSV, and ASPV, which were detected at rates of 100% (175 of 175), 78% (136 of 175), and 71% (125 of 175), respectively. These three latent viruses are widely prevalent in cultivated apple across North America and are known to be the most prevalent viruses infecting apple trees in commercial orchards in New York [3,27]. Five additional viruses, i.e., CCGaV, AGCaV, ARWV2, TRSV, and ToRSV, as well as one viroid, AHVd, were detected in one or more of the trees tested (Table 3). The four trees in which TRSV or ToRSV was detected were located in a single row on the westernmost edge of the study orchard, suggesting infection may have occurred via dagger nematodes of the species complex *Xiphinema americanum* present in the soil prior to study orchard establishment. The other ten viruses and viroids detectable by this methodology (i.e., ALV1, ApMV, ARWV1, CiVA, PpPV2, PrVT, AFCVd, ADFVd, ASSVd, and PBCVd) were not identified in the samples tested in this study.

### 3.2. Evaluation of Seed Transmission of ACLSV, ASGV, and ASPV in Malus domestica

A total of 3283 seeds resulting from the open pollination of 175 maternal apple trees were treated with sodium hypochlorite, stratified, and germinated under greenhouse conditions for virus testing. The germination rate (87%, 2863 of 3283) and viability rate of seedlings after transplanting (94%, 2701 of 2863) were high at three weeks post germination. Seedlings were tested for ACLSV, ASGV, and ASPV by multiplex RT-PCR at the 6–8 true leaf stage to assess the occurrence of seed transmission. Of 1121 progeny of 56 unique ACLSV-infected maternal apple trees, none was found to be positive for ACLSV (Table 4). Similarly, of the 1412 and 1035 progeny seedlings of 68 unique ASGV- and 51 unique ASPV-infected maternal apple trees, none was found to be positive for either ASGV or ASPV, respectively (Table 4). These results indicated that seed transmission did not occur in this study.

### 3.3. Evaluation of Seedborne Nature of ACLSV, AGCaV, ARWV2, ASGV, ASPV, and CCGaV in Malus domestica

Fruits from four trees known to be infected with ASPV in the experimental orchard were collected for the testing of peel, flesh, and immature seeds for ASPV by multiplex RT-PCR. Specific DNA amplicons were obtained for each of the three tissue types, demonstrating that ASPV is present at detectable levels throughout apple fruits (100%, 12 of 12). Moreover, ASPV was detected in total RNA extracted from the seeds of four storebought ‘Gala’ apple fruits by multiplex RT-PCR (100%, 4 of 4). However, ASPV was not detectable in seeds of these fruits after a sodium hypochlorite treatment (0%, 0 of 4). This result suggested that the virus was present in or on the seedcoat. To test this hypothesis with fruits produced by trees in the experimental orchard, RNA was isolated from untreated seeds representing the progeny of 49 virus-infected trees. ACLSV, AGCaV, ARWV2, ASGV, ASPV, and CCGaV were detected by RT-PCR or RT-qPCR at rates of 93% (38 of 41), 40% (8 of 20), 20% (1 of 5), 96% (47 of 49), 92% (35 of 38), and 84% (27 of 32), respectively (Table 4). These results demonstrated the potential for all six of these viruses to be seedborne in *M. domestica*.

## 4. Discussion

Seed transmission represents an important route of virus transmission with at least one quarter of plant viruses known to be transmitted through the seed of at least one of their hosts. This proportion is expected to grow with the use of increasingly sensitive detection methods enabling the identification of novel seedborne viruses, as well as with the detection of seedborne viruses in asymptomatic hosts [27,28,29,30]. Two routes of seed transmission have been identified. Some viruses are seedborne, wherein the virus is present as a contaminant on the seedcoat or is carried within the seedcoat or endosperm but is not found in the embryo itself [28,29,31]. These include tobacco mosaic virus in tomato [32], rice yellow mottle virus in rice [33], and pepino mosaic virus (PepMV) in tomato [34,35]. Other viruses are carried within the tissues of the embryo itself, which is achieved either through direct infection from the maternal plant or via infected pollen [28,31].

Embryo transmission is more common and has been reported for many plant viruses, including members of at least 20 genera [28]. Proportions of infected seeds vary widely based on many factors, including virus strain, host species, host resistance, host developmental stage at the time of infection, seed age, virus stability, and environmental factors [28]. Host seedlings may be infected as a direct result of embryo infection, but seedlings arising from uninfected embryos may also be infected during germination by virus present in infected seedcoat or endosperm or by virus present as a seedcoat contaminant, as in cucumber green mottle mosaic virus in cucumber and watermelon [36], PepMV in tomato [34,35], and tobacco mosaic virus and tomato brown rugose fruit virus in tomato and pepper [37,38].

Seed transmission is an important facet of plant virus epidemiology and, therefore, is important to consider when shaping phytosanitary measures [28]. The aim of this study was to evaluate whether the major viruses of pome fruit trees could be seedborne or seed transmitted in commercial apple cultivars such as ‘Brookfield Gala’, ‘Honeycrisp’, and ‘Royal Red Honeycrisp’. Our findings demonstrated that six apple viruses (i.e., ACLSV, AGCaV, ARWV2, ASGV, ASPV, and CCGaV) can indeed be seedborne, some at high rates. They also suggested that ACLSV, ASGV, and ASPV are likely not seed transmitted or are seed transmitted at very low rates via sterilized seeds of *M. domestica*.

Similarly to the work of Li et al. [21] on the transmission of ACLSV, ASGV, and ASPV in two pear rootstock species, we found that these three viruses can be detected from the seedcoats of seeds extracted from fruits of infected maternal trees. The rates at which these three viruses were detected in untreated seeds were higher in our experiments (92.7%, 95.9%, and 92.1%, respectively) vs. the rates reported by Li et al. [21] (40.4%, 73.9%, and 21.2%, respectively). These differences may reflect biological differences between the host species studied (*P. betulifolia* and *P. calleryana* vs. *M. domestica*), genetic divergence among virus isolates, or differences in the virus detection methodologies employed. Regardless, it is clear that these three viruses of the family *Betaflexiviridae* can be seedborne in pome fruit trees at high rates.

In contrast to the findings of Li et al. [21], we were unable to detect ACLSV, ASGV, or ASPV in the seedling progeny of infected maternal trees at the 6–8 true leaf stage (0%, 0%, and 0% vs. 15.6%, 9.3%, and 2.7% reported by Li et al. [21], respectively). It is possible that these differences are attributable to biological differences between the two host species under consideration, such as differences in the abilities of the evaluated viruses to successfully invade *Pyrus* and *Malus* embryos. An example of this phenomenon was reported for raspberry bushy dwarf virus, which is capable of seed transmission in red raspberry, but is incapable of seed transmission in *Nicotiana benthamiana* due to an inability to invade the embryo, though the mechanism preventing embryo invasion is not known [39]. Although the evolutionary distance between the *Malus* and *Pyrus* genera is certainly much smaller than between red raspberry (*Rubus*) and *Nicotiana*, the possibility of differential virus-host interactions within *Malus* and *Pyrus* cannot be ruled out. Additionally, a major difference between the experimental designs of the two studies was the sodium hypochlorite seed surface sterilization treatment utilized in our study. The high rates of virus detection in untreated seed and the absence of virus detection in both treated seed and in the progeny derived from treated seed reported here indicated that seed transmission via infected embryonic tissue is unlikely in *M. domestica* or may occur at rates below the limit of detection of our RT-PCR assays. For example, PepMV, initially believed not to be seed transmitted in tomato, was later determined to be seed transmitted at a rate of 0.005–0.057% following a large-scale screen of over 87,000 plants [34,35].

The successful detection of ACLSV, ASGV, and ASPV in the seedling progeny of infected maternal trees at the 6–8 true leaf stage reported by Li et al. [21] may reflect infection by seedborne virus during germination or may indeed represent embryo infection in *P. betulifolia* and *P. calleryana*. Despite the lack of virus detection in *M. domestica* progeny derived from treated seed reported in the current study, transmission of one or multiple of these viruses may still be possible in *M. domestica* via infection during germination of untreated seeds, which was not evaluated in this study. This important question should be addressed in a future study.

The infection mechanism(s) operating in this system are unclear and were not evaluated in our study. Our detection of ASPV in the peel and flesh of apple fruits produced by infected maternal trees demonstrates that this virus is clearly present in maternal tissues surrounding the seeds, and therefore could also be present in the maternally derived seedcoat or nucellus as well. However, it is also possible that the biparental tissues (i.e., the embryo and endosperm) could be infected via infected pollen, which was not evaluated here.

The objective of this study was to determine whether the major viruses known to infect pome fruit trees are seedborne or seed transmitted in one of the world’s most widely grown fruit crops, *M. domestica*. Our findings support our hypothesis that AGCaV, ACLSV, ARWV2, ASGV, ASPV, and CCGaV are seedborne in commercial apple cultivars, but refute our hypothesis that ACLSV, ASGV, and ASPV are seed transmitted in this system, at least via treated seeds. Further studies should be undertaken to determine whether seed transmission of major apple-infecting viruses can occur in untreated seeds of *M. domestica.* If this is found to occur, seed treatments should be further evaluated and considered by breeders, policymakers, and regulators to facilitate the development of virus-free apple trees and the safe exchange of apple germplasm.

## Figures and Tables

**Table 1:** Viruses and viroids included in testing of apple maternal trees via multiplex PCR-based amplicon sequencing.

Family	Genus	Virus or Viroid	Abbreviation
*Avsunviroidae*	*Pelamoviroid*	Apple hammerhead viroid	AHVd
*Betaflexiviridae*	*Capillovirus*	Apple stem grooving virus	ASGV
	*Foveavirus*	Apple green crinkle-associated virus	AGCaV
		Apple stem pitting virus	ASPV
	*Tepovirus*	Prunus virus T	PrVT
	*Trichovirus*	Apple chlorotic leaf spot virus	ACLSV
*Bromoviridae*	*Ilarvirus*	Apple mosaic virus	ApMV
*Tombusviridae*	*Luteovirus*	Apple luteovirus 1	ALV1
*Partitiviridae*	*Alphapartitivirus*	Pyrus pyrifolia partitivirus 2	PpPV2
*Phenuiviridae*	*Coguvirus*	Citrus concave gum-associated virus	CCGaV
		Citrus virus A	CiVA
	*Rubodvirus*	Apple rubbery wood virus 1	ARWV1
		Apple rubbery wood virus 2	ARWV2
*Pospiviroidae*	*Apscaviroid*	Apple dimple fruit viroid	ADFVd
		Apple fruit crinkle viroid	AFCVd
		Apple scar skin viroid	ASSVd
		Pear blister canker viroid	PBCVd
*Secoviridae*	*Nepovirus*	Tobacco ringspot virus	TRSV
		Tomato ringspot virus	ToRSV

**Table 2:** Primers and probes used in RT-PCR and RT-qPCR assays in this study.

Target	Type	Oligo Name	Sequence (5′–3′)	Target Region	Reference
RT-PCR					
ACLSV	Primer	ACLSV-F	TTCATGGAAAGACAGGGGCAA	Coat protein	[24]
	Primer	ACLSV-R	AAGTCTACAGGCTATTTATTATAAGTCTAA		
ASGV	Primer	ASGV-F	GCCACTTCTAGGCAGAACTCTTTGAA	Coat protein	[24]
	Primer	ASGV-R	AACCCCTTTTTGTCCTTCAGTACGAA		
ASPV	Primer	ASPV-F	ATGTCTGGAACCTCATGCTGCAA	Coat protein	[24]
	Primer	ASPV-R	TTGGGATCAACTTTACTAAAAAGCATAA		
nad5	Primer	nad5-F	GATGCTTCTTGGGGCTTCTTGTT	nad5 mRNA	[24]
	Primer	nad5-R	CTCCAGTCACCAACATTGGCATAA		
RT-qPCR					
AGCaV	Primer	AGCaV-RT-F	GCVAAYGCCACAAGCAAA	Coat protein	[23]
	Primer	AGCaV-RT-R	GYCCAAYYTTTCCCCCAGTGA		
	Probe	AGCaV-RT-P	CAAGCTGTYAACTTTGGY		
ARWV2	Primer	ARWV2-RT-F	GAGATTCCAAGCTATTTCATAAGCATAA	RdRP (Segment L)	[25]
	Primer	ARWV2-RT-R	TGATAAAAGGCACACAAGTTGCA		
	Probe	ARWV2-RT-P	ATCCCCAGTGCTGAAA		
ASPV	Primer	ASPV-RT-F	GAGGAGGTTGGCACGATGATC	Coat protein	[26]
	Primer	ASPV-RT-R	GCAGCATGAGGTTCCAGACAT		
	Probe	ASPV-RT-P	AACTGAAGGGTGCACTTTGAGGCA		
CCGaV	Primer	CCGaV-RT-F	GCCTTTCTTCGTACATGTGATGAG	RdRP (RNA1)	[25]
	Primer	CCGaV-RT-R	GGCAAACTGTATGGGAAAGCTAA		
	Probe	CCGaV-RT-P	TGTGGAAATGCAAGAGA		

Abbreviations: ACLSV, apple chlorotic leaf spot virus; AGCaV, apple green crinkle-associated virus; ARWV2, apple rubbery wood virus 2; ASGV, apple stem grooving virus; ASPV, apple stem pitting virus; CCGaV, citrus concave gum-associated virus; F, forward; mRNA, messenger RNA; nad5, NADH dehydrogenase subunit 5; P, probe; R, reverse; RdRP, RNA-dependent RNA polymerase; RT-PCR, reverse transcription polymerase chain reaction; RT-qPCR, quantitative RT-PCR; Segment L, large segment.

**Table 3:** Viruses and viroids detected in 175 maternal apple trees via multiplex PCR-based amplicon sequencing.

Virus or Viroid	Trees Infected	% Trees Infected
Apple stem grooving virus	175	100
Apple chlorotic leaf spot virus	136	77.7
Apple stem pitting virus	125	71.4
Citrus concave gum-associated virus	106	60.6
Apple hammerhead viroid	104	59.4
Apple green crinkle-associated virus	56	32.0
Apple rubbery wood virus 2	25	14.3
Tobacco ringspot virus	3	1.7
Tomato ringspot virus	1	0.6

**Table 4:** Viruses detected in seeds and progeny seedlings of infected maternal apple trees.

	Seeds			Seedlings		
Virus	N ^a^	Positive ^b^	Incidence (%)	N ^a^	Positive ^b^	Incidence (%)
ACLSV	41	38	92.7	1121	0	0
ASGV	49	47	95.9	1412	0	0
ASPV	38	35	92.1	1035	0	0
AGCaV	20	8	40.0	nt	na	na
ARWV2	5	1	20.0	nt	na	na
CCGaV	32	27	84.4	nt	na	na

^a^ Number of seeds or seedlings tested in RT-PCR. ^b^ Number of positive seeds or seedlings in RT-PCR. Abbreviations: ACLSV, apple chlorotic leaf spot virus; ASGV, apple stem grooving virus; ASPV, apple stem pitting virus; AGCaV, apple green crinkle-associated virus; ARWV2, apple rubbery wood virus 2; CCGaV, citrus concave gum-associated virus; na, not applicable; nt, not tested.

## Data Availability

The data presented in this study are available in the article.
